# Supplementary figures and images for: Histone H1x in mouse ventral hippocampus associates with, but does not cause behavioral adaptations to stress
Source: Transl Psychiatry. 2024 Jun 5;14:239. doi: 10.1038/s41398-024-02931-x (PMC11150540; doi:10.1038/s41398-024-02931-x)

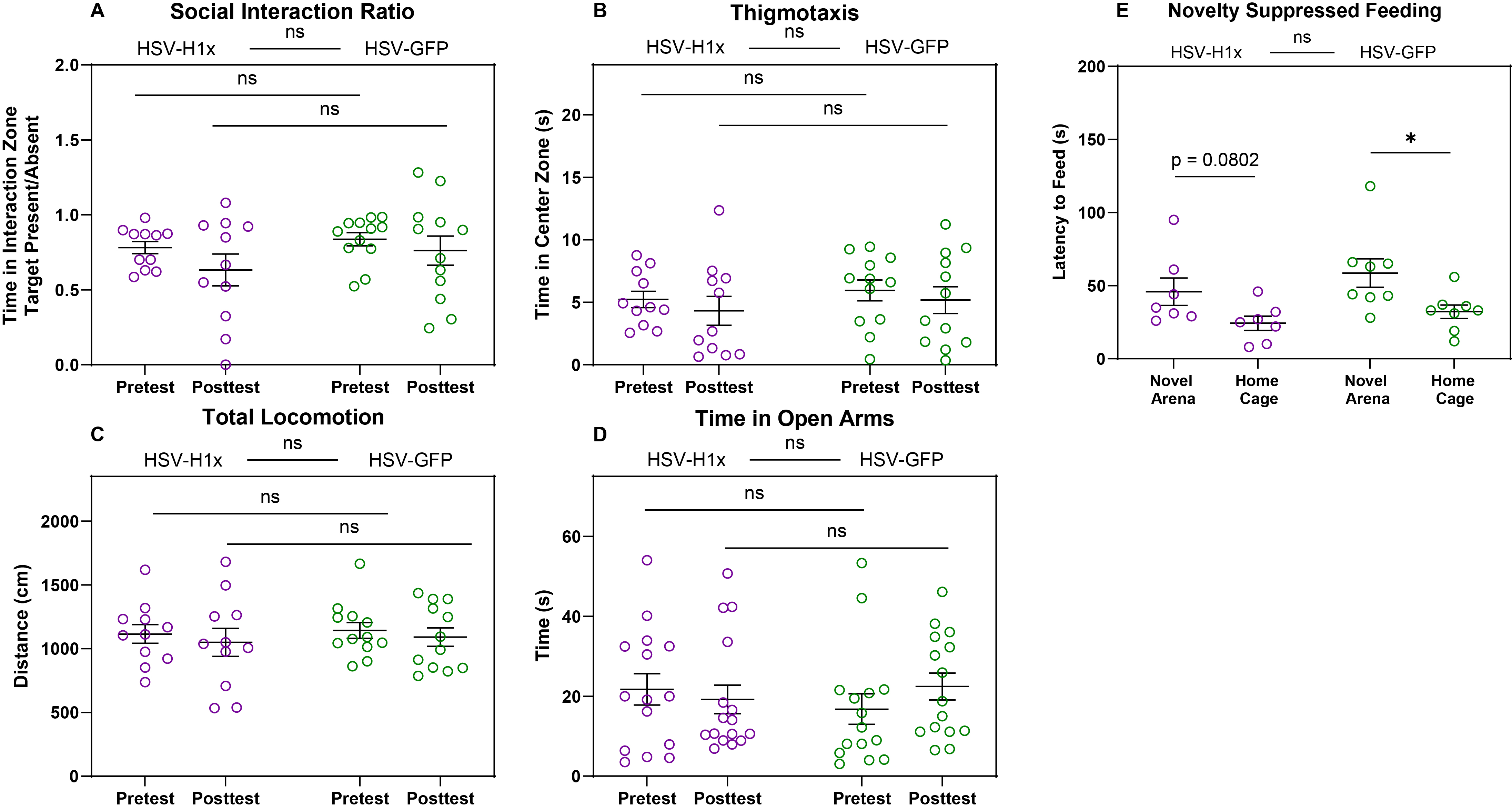

Supplement: Supplementary file 1 — Supplemental Figure 1 [file 41398_2024_2931_MOESM1_ESM.jpg]

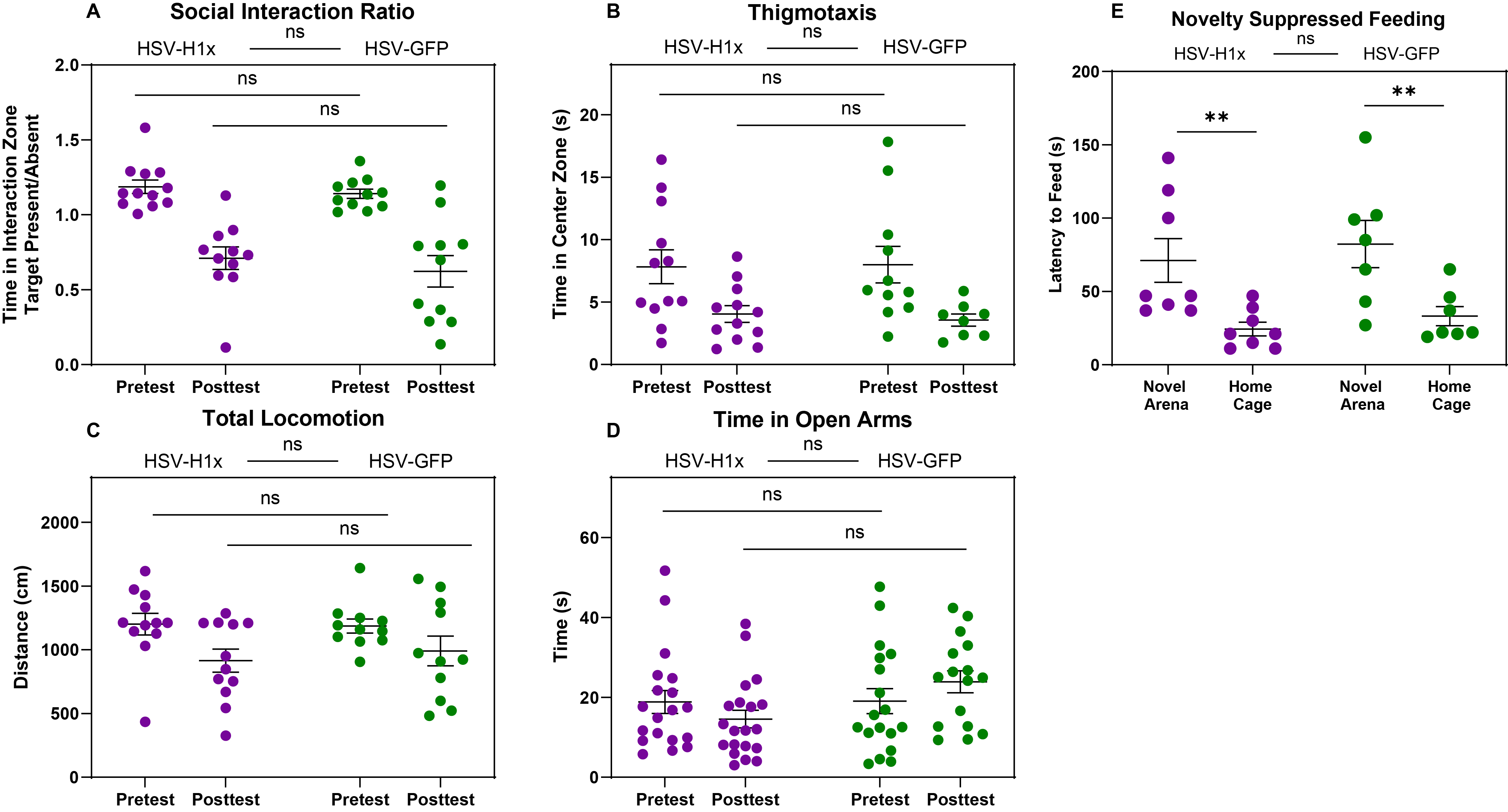

Supplement: Supplementary file 2 — Supplemental Figure 2 [file 41398_2024_2931_MOESM2_ESM.jpg]

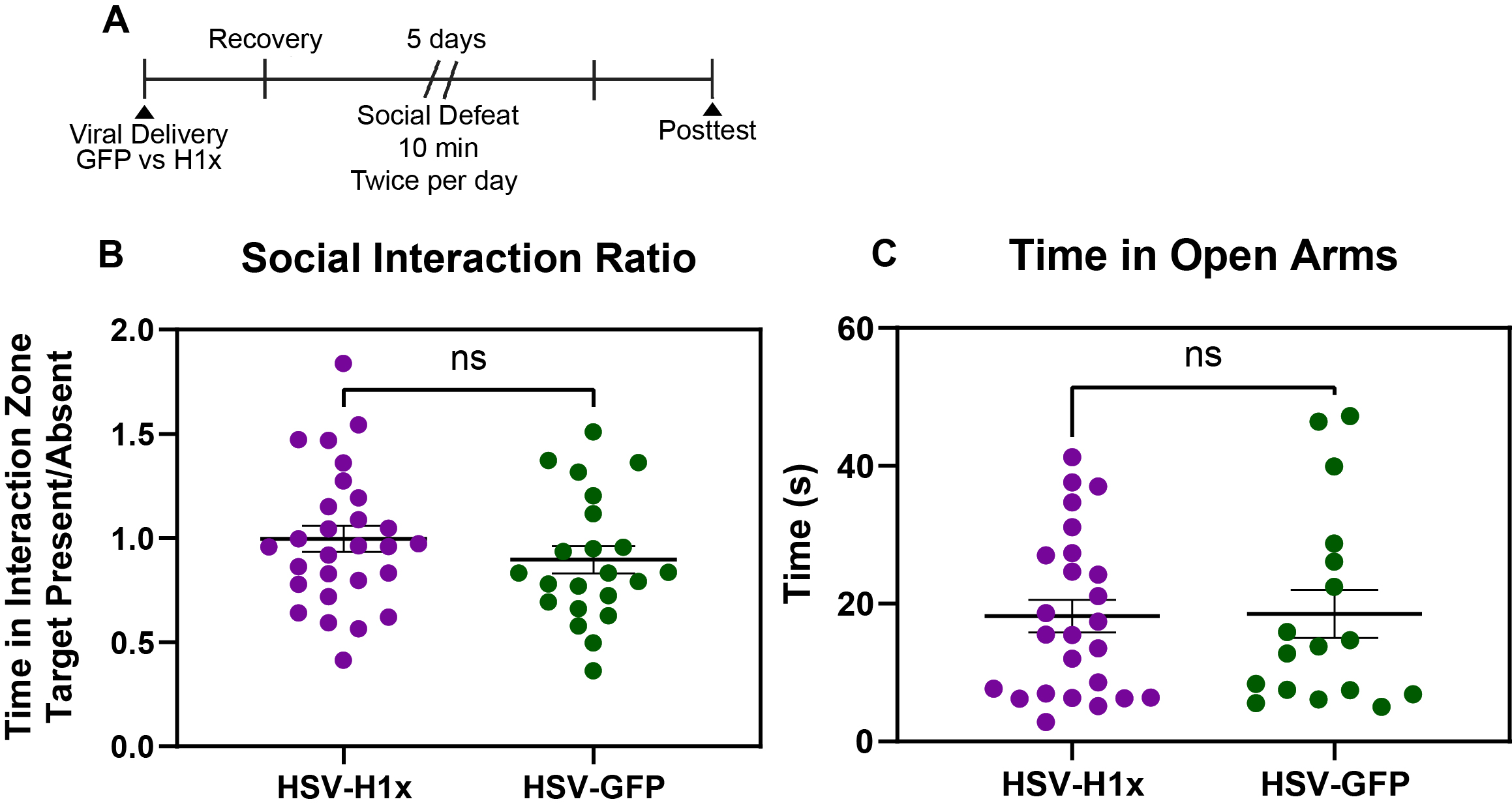

Supplement: Supplementary file 3 — Supplemental Figure 3 [file 41398_2024_2931_MOESM3_ESM.jpg]

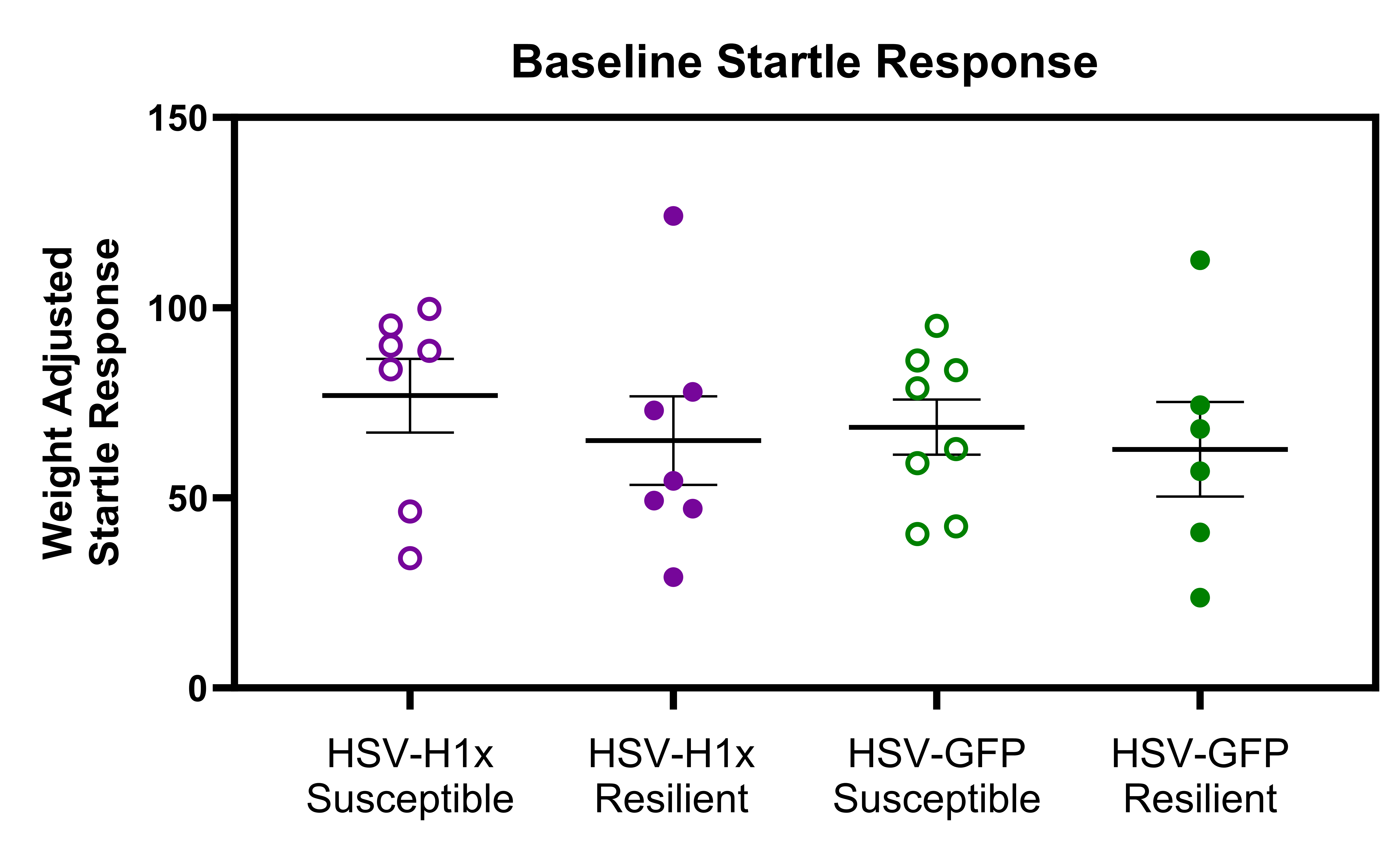

Supplement: Supplementary file 4 — Supplemental Figure 4 [file 41398_2024_2931_MOESM4_ESM.tif]

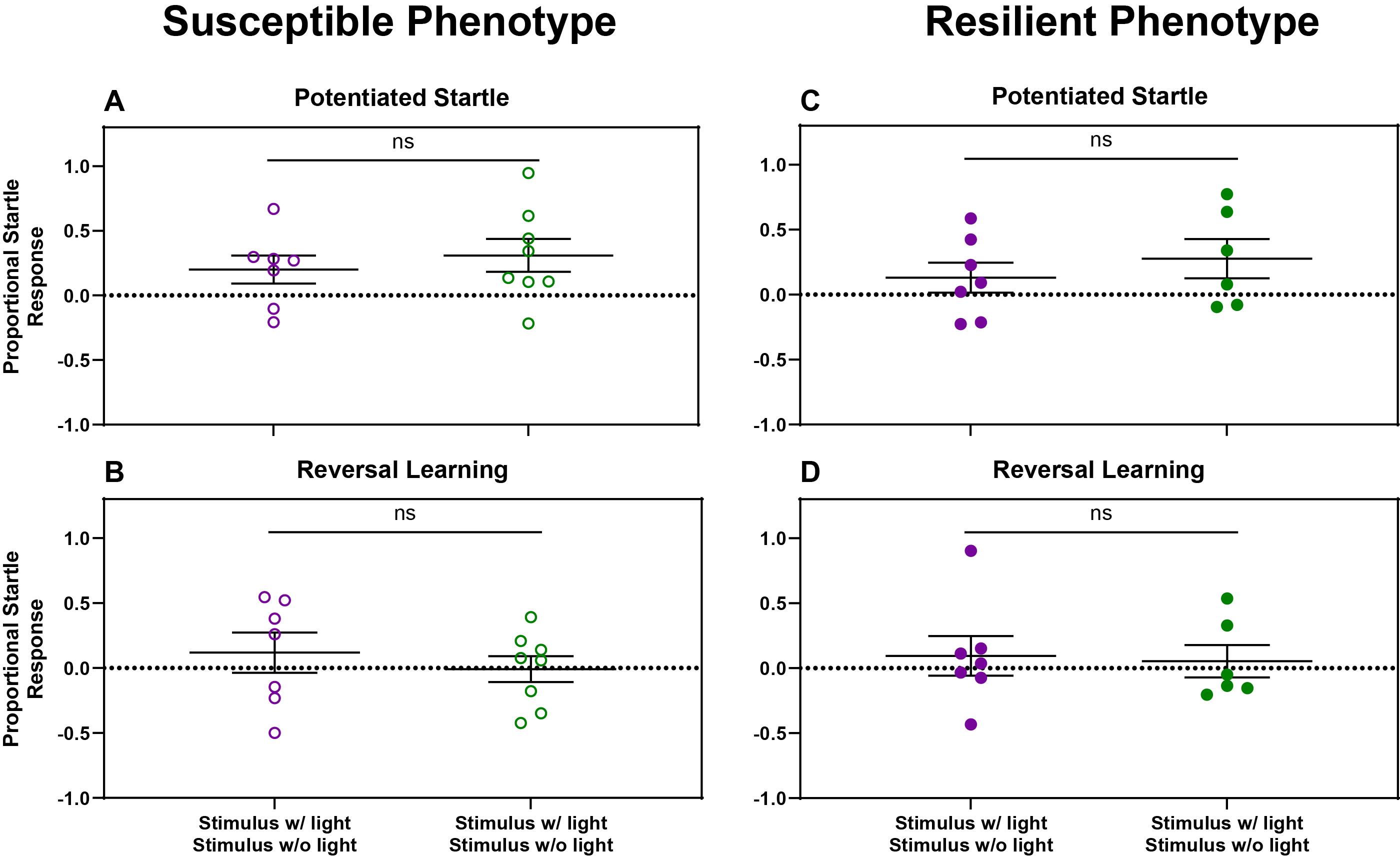

Supplement: Supplementary file 5 — Supplemental Figure 5 [file 41398_2024_2931_MOESM5_ESM.jpg]
