# Supplementary material for: Histone H1x in mouse ventral hippocampus associates with, but does not cause behavioral adaptations to stress
Source: Transl Psychiatry. 2024 Jun 5;14:239. doi: 10.1038/s41398-024-02931-x (PMC11150540; doi:10.1038/s41398-024-02931-x)
Supplement: Supplementary file 6 — Supplemental figure captions [file 41398_2024_2931_MOESM6_ESM.docx]

**Supplemental Figure 1: Behavioral results of intra-hippocampal overexpression of H1x in stress-susceptible mice.** No change in behaviors were observed between mice treated with HSV-H1x versus HSV-GFP (Two-way ANOVA with Bonferroni’s correction, p > 0.05).

**Supplemental Figure 2: Behavioral results of intra-hippocampal overexpression of H1x in stress-resilient mice.** No change in behaviors were observed between mice treated with HSV-H1x versus HSV-GFP (Two-way ANOVA with Bonferroni’s correction, p > 0.05).

**Supplemental Figure 3: Accelerated social defeat after delivery of HSV-H1x did not alter behavioral adaptations to stress experience.** (A) Experimental timeline. Accelerated twice per day social defeats were completed as previously described, with defeats spaced 6 hours apart. Behavioral testing involved social interaction and elevated plus maze behaviors. (B) No significant difference in social interaction ratios was observed between mice treated with HSV-H1x versus HSV-GFP (Two-tailed t-test, p > 0.05). Further, there was no change in the proportion of mice found to be susceptible to stress between either virus treatment group (Fisher’s exact test, p > 0.05). (C) No change in elevated plus maze exploration was observed between viral groups (Two-tailed t-test, p > 0.05).

**Supplemental Figure 4: Susceptible and resilient mice treated with HSV-H1x versus HSV-GFP do not show differences in baseline acoustic startle behaviors.** Following viral overexpression of H1x versus GFP, no differences in startle response to acoustic stimuli were observed between susceptible or resilient mice treated with either HSV-H1x or HSV-GFP (One-way ANOVA, p > 0.05).

**Supplemental Figure 5: Fear-potentiated startle behaviors are not driven by a single stress-induced phenotype.** Separating data by pretest phenotype does not reveal phenotype-specific deficits in either acquisition or reversal of potentiated startle (Two-way ANOVA with Bonferroni’s correction, p > 0.05).
